# Kinetics of gene expression and bone remodelling in the clinical phase of collagen-induced arthritis

**DOI:** 10.1186/s13075-015-0531-7

**Published:** 2015-03-05

**Authors:** Katja CM Denninger, Thomas Litman, Troels Marstrand, Kristian Moller, Lars Svensson, Tord Labuda, Åsa Andersson

**Affiliations:** Department of Drug Design and Pharmacology, Faculty of Health and Medical Sciences, University of Copenhagen, Universitetsparken 2, Copenhagen, Ø DK-2100 Denmark; Disease Pharmacology/Molecular Biomedicine, LEO Pharma A/S, Industriparken 55, Ballerup, DK-2750 Denmark

## Abstract

**Introduction:**

Pathological bone changes differ considerably between inflammatory arthritic diseases and most studies have focused on bone erosion. Collagen-induced arthritis (CIA) is a model for rheumatoid arthritis, which, in addition to bone erosion, demonstrates bone formation at the time of clinical manifestations. The objective of this study was to use this model to characterise the histological and molecular changes in bone remodelling, and relate these to the clinical disease development.

**Methods:**

A histological and gene expression profiling time-course study on bone remodelling in CIA was linked to onset of clinical symptoms. Global gene expression was studied with a gene chip array system.

**Results:**

The main histopathological changes in bone structure and inflammation occurred during the first two weeks following the onset of clinical symptoms in the joint. Hereafter, the inflammation declined and remodelling of formed bone dominated.

Global gene expression profiling showed simultaneous upregulation of genes related to bone changes and inflammation in week 0 to 2 after onset of clinical disease. Furthermore, we observed time-dependent expression of genes involved in early and late osteoblast differentiation and function, which mirrored the histopathological bone changes. The differentially expressed genes belong to the bone morphogenetic pathway (BMP) and, in addition, include the osteoblast markers integrin-binding sialoprotein (*Ibsp*), bone gamma-carboxyglutamate protein (*Bglap1*), and secreted phosphoprotein 1 (*Spp1*). Pregnancy-associated protein A (*Pappa*) and periostin (*Postn*), differentially expressed in the early disease phase, are proposed to participate in bone formation, and we suggest that they play a role in early bone formation in the CIA model. Comparison to human genome-wide association studies (GWAS) revealed differential expression of several genes associated with human arthritis.

**Conclusions:**

In the CIA model, bone formation in the joint starts shortly after onset of clinical symptoms, which results in bony fusion within one to two weeks. This makes it a candidate model for investigating the relationship between inflammation and bone formation in inflammatory arthritis.

**Electronic supplementary material:**

The online version of this article (doi:10.1186/s13075-015-0531-7) contains supplementary material, which is available to authorized users.

## Introduction

Inflammatory arthritis is characterised by joint destruction, often resulting in severe distortion of the joint architecture, and leading to impaired function of the joints. The balance between anabolic and catabolic bone remodelling, however, differs between the inflammatory arthritic diseases rheumatoid arthritis (RA), psoriatic arthritis (PsA), and ankylosing spondylitis (AS). While RA primarily is an erosive disease, bone erosion and bone formation co-exist in patients with PsA and AS [1]. Why the bone responds differently to an inflammatory insult is not clarified, but may be important in directing future treatment strategies.

Collagen-induced arthritis (CIA) is a model for RA both due to its construct validity, including autoimmune reaction to collagen type II and MHC class II dependency, and due to its face validity, that is severe bone destruction. Moreover, disease development ends in bony fusion (ankylosis) of the destroyed joints, which could make it a relevant model for bone-forming inflammatory arthritic diseases.

Bone formation in CIA has been described as starting two to four weeks after onset of arthritis, but mainly playing a role in the healing state two to three months after onset [2]. However, in a kinetic study of new bone formation in CIA and adjuvant-induced arthritis (AIA) in rat, periosteal proliferation was reported as early as day 3 (CIA) and day 5 (AIA) after onset of arthritis, and new bone formation peaked at day 27 in both models [3].

Bone formation may occur directly within the mesenchyme (intramembranous) or via a cartilage scaffold (endochondral ossification). In both processes, the extracellular matrix (ECM) is mineralised. In particular two pathways have been implicated in osteoblast differentiation and bone formation: the bone morphogenetic protein (BMP) signalling pathway, of which several proteins induce ectopic bone formation [4], and the wingless-type (WNT) signalling pathway [5].

With the aim to understand the molecular mechanisms linking inflammation with new bone formation, the present study was initiated to histologically characterise bone remodelling in the CIA model throughout the disease course, and to relate this to gene expression. To minimise variation between the data and enable correlation to clinical duration of arthritis, we used tarsal joints with a clinical score of 3. By grouping the tarsal joints according to onset and duration of symptoms, we show that the process of bone formation runs in parallel with the local inflammatory process in the joint, with a concomitant differential expression of genes involved in bone remodelling.

## Methods

### Animal model

Arthritis was induced in male DBA/1JBom mice by intradermal injection of 100 μl chicken collagen type II (CII) (2 mg/ml) (Sigma-Aldrich, St Louis, MO, USA) in complete Freund’s adjuvant (CFA) (0.5 mg/ml) (Sigma-Aldrich) on day 1 and boosted on day 21 with CII in incomplete Freund’s adjuvant (IFA) (Sigma-Aldrich). The control group received CFA and IFA without collagen, respectively. The mice were scored for onset, severity and duration of arthritis in the tarsal joints. Severity in this joint was scored from 0 (no erythema or swelling) to 3 (severe erythema and swelling). Tarsal joints were collected for histology and gene expression analysis. The mice were, in addition, assigned a total score for all four paws (with a theoretical maximum of 24 points for all paws). This additional scoring system was used to judge when the animals should be euthanized as required by Danish legislation (score exceeding 10 points). The mice were housed under standard conditions, and provided pellets and water *ad libitum*. The Danish Ethics Authorities ‘The Animal Experiments Inspectorate’ approved the studies (permit number: 2008/561 − 1532).

### Histology

Fifty-two tarsal joints were collected for histology. The tarsal joints were divided into groups according to duration of arthritis in the joint: day 0 to 3, day 4 to 7, week 1 to 2, > 2 weeks, and > 2 weeks with declining inflammation (‘declined’). In the group ‘declined’ the joints previously had a clinical score of 3 for minimum two weeks, after which the clinical score had declined > 1 score at the time the joint was sampled. For all other joints, the clinical score was 3 at the sampling time. For number of joints per time-point, see Table S2A in Additional file 1. The joints were fixed in formalin and decalcified in 10% Na-EDTA for two to three weeks.

Haematoxylin and eosin-stained sections were assigned a score for bone erosion, bone formation, synovitis, tendinitis, and peritendinitis. Bone remodelling was judged by the degree of modulation of joint structure, presence of osteoclasts (OCs) and osteoblasts (OBs), and formation of cartilage and woven bone (Figure S1 in Additional file 2). Synovitis, tendinitis and peritendinitis were scored by the degree of infiltrating cells and fibrosis. All features were scored from 0 (no pathologic changes) to 4 (severe pathologic changes).

### RNA extraction and microarray analysis

Three tarsal joints were sampled per group (twelve joints in total). For the group ‘More than two weeks, with declined inflammation’ the joints had had a clinical score of 3 for minimum two weeks, after which the clinical score had declined > 1 score at the time for sampling. For all other joints, the clinical score was 3 when sampled (Table S2B in Additional file 1). The twelve joints were compared to three joints from non-immunised control animals. The joints were processed and analysed separately (unpooled). Skin, visible muscle tissue and fur were removed and tarsal joints collected by transverse sectioning of the metatarsus bones and the tibia/fibula under the fur border. This procedure ensured that all joints in the tarsus were included in each sample. Except for the skin and visible muscle, all other soft tissues were included in the sample. The joints were subsequently snap-frozen in liquid N_2_ and stored at −80°C. RNA was extracted using the *mir*Vana^™^ miRNA isolation kit (Ambion, Exiqon A/S, Vedbæk Denmark), amplified and labelled using the Pico amplification kit (Nugen Technologies, San Carlos, CA, USA), according to the manufacturers’ instructions, followed by hybridisation to Mouse Gene 1.0ST microarrays (Affymetrix, Santa Clara, CA, USA).

The quality of the RNA was evaluated using the RNA integrity number (RIN), and only samples with RIN >8.3 were included for further analysis.

Arrays were normalized using RMA background correction and quantile normalisation in R v.2.15 (Additional file 3). Significance of differentially expressed genes was assessed by analysis of variance (ANOVA), and adjusted for multiple testing by estimating false discovery rates (FDR). Data visualisation was performed in Qlucore Omics Explorer v.2.2 (Qlucore AB, Lund, Sweden). The expression data are deposited in Gene Expression Omnibus [6]; accession number: Series record GSE61140).

Functional analysis and network representation of differentially expressed genes was performed in Ingenuity Pathway Analysis (IPA, Ingenuity^™^ Systems). Gene expression levels are indicated in the IPA networks as red (upregulated) or green (downregulated) in comparison to non-induced controls.

The differentially regulated genes assigned to Bio Functions of bone and skeleton, were used to compile a comprehensive list of genes potentially involved in bone remodelling in the CIA model.

### Quantitative PCR

For qPCR analysis of *Bmp1*, RNA (90 ng per reaction) was reverse transcribed to cDNA using the High Capacity cDNA Reverse Transcription kit (Applied Biosystems, Carlsbad, CA, USA). qPCR was performed with TaqMan Universal PCR Mastermix (Applied Biosystems, USA) and primer-assays Bmp1 Mm00802225_m1 and Gapdh Mm99999915_g1 (Applied Biosystems, USA). PCR was initiated at 50°C for 2 minutes and 95°C for 10 minutes, and the cycling conditions were 95°C for 15 seconds and 60°C for 1 minute (40 cycles). For the remaining 14 genes (Table S4 in Additional file 4), qPCR analysis was performed by AROS Applied Biotechnology A/S (Aarhus, Denmark). cDNA was prepared from 100 ng RNA/sample using the High Capacity cRNA Reverse Transcription kit (Applied Biosystems, USA) and the qPCR reaction was performed with TaqMan^™^ Universal PCR Master Mix (Applied Biosystems, USA). Assay IDs for the specific primer/probe sets (Life Technologies, Carlsbad, CA, USA) are listed in Table S4 in Additional file 4. Samples were analysed as triplicates and normalized by subtraction of the sample mean Ct value from that of the mean Ct value of the housekeeping gene Gapdh.

### Statistics

Histological scoring results were assessed using Kruskall-Wallis test followed by a Dunn’s multiple comparisons test. qPCR results were analysed by ANOVA, followed by Bonferroni’s multiple comparison test, and compared by linear regression to array results. The significance of the correlation coefficients was tested using Student's *t*-distribution. All statistical analyses were performed in GraphPad Prism v.5 (GraphPad Software, San Diego, CA, USA), with the statistical unit being one joint from each animal included.

## Results

### Development of arthritis and histopathology of CIA from onset to decline of clinical symptoms

After immunization with chicken CII, mean onset of arthritis was observed on day 31 ± 8.8. Mean score of arthritis peaked six weeks after boost with a mean score of 4.6 ± 1.8. In order to study local arthritis development, onset and severity of arthritis in the tarsal joint was recorded in detail.

For the histological study, a total of 52 tarsal joints were used. Fourteen were sampled between day 0 and 4, ten between day 4 and 7, eleven between week 1 and 2, and seventeen after week 2 of clinical inflammation onset. After week 2, twelve samples showed declining clinical symptoms. Little variation in the appearance of synovitis, peritendinitis, and bone remodelling was noted within the groups. The data are in accordance with a previously published study showing occurrence of bony spur formation early after disease onset [3].

**Figure 1:**
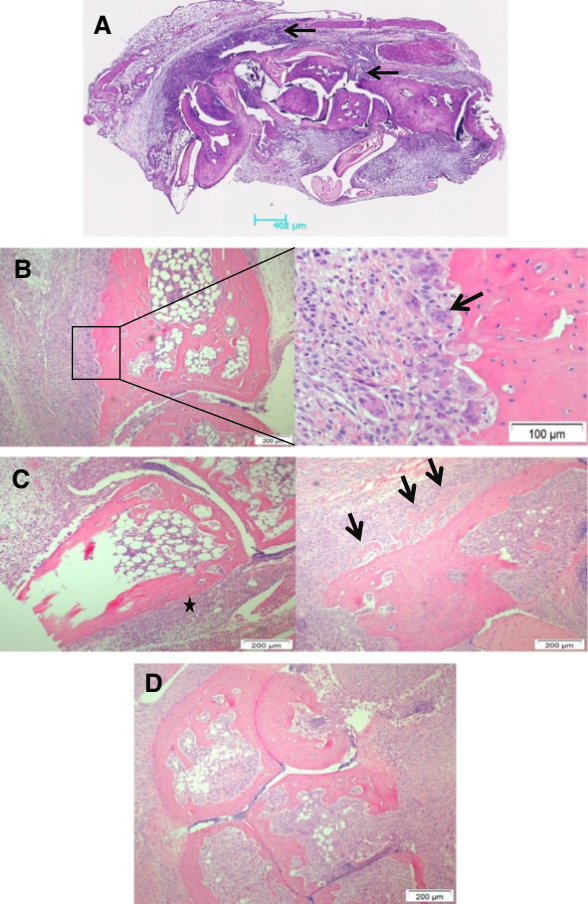
**Histopathology of tarsal joints day 0 to 7. A)** Day 0 to 4: overview of inflammation in the tarsal joint showing equal severity of inflammation in peripheral tissues and synovium (arrows). **(B-C)** Day 0 to 4: bone erosion and OC activity (arrow in B, day 3) dominates. Proliferation and collagen deposition takes place (star in C, day 0) in the periosteum and results in osteoid deposition (arrows in C, day 3). **(D)** Day 4 to 7: the joint architecture is severely distorted (day 7). OC, osteoclast.

**Day 0 to 4 after clinical disease onset:** The infiltration of inflammatory cells was equally pronounced when comparing the synovium (mean score 3.8 +/−0.4) and the peritendon (mean score 3.9+/−0.3) (Figure 1A). In 10/14 joints, fibroblast activity and fibrosis indicated chronic inflammation, while 4/14 joints had no signs of chronicity. Bone pathology was dominated by erosion (11/14 joints), initiated where the peritendon and synovium are attached to, or in close proximity to the bone, while the articular cartilage remained intact. Bone erosion was severe as judged by the presence of multinucleated osteoclasts (OC) and the extent of structural damage of the cartilage and bone tissue (Figure 1B). However, both OB proliferation, and collagen deposition, was observed in the periosteum and seemed, from visible staining ability, to have progressed into secretion of osteoid-like matrix in 3/14 joints (Figure 1C).

**Day 4 to 7 after clinical disease onset:** Severe fibrosis and synovitis approaching the articular cartilage, and resulting in erosion of the articular cartilage, was observed (Figure 1D). In areas of less severe inflammation, pronounced OB activity and onset of endochondral ossification was noted as judged from the appearance of hypertrophic cartilage. Bone erosion (mean score of 3.1+/−1.3) and bone formation (2.2+/−1.1), was observed in 9/10 joints.

**Figure 2:**
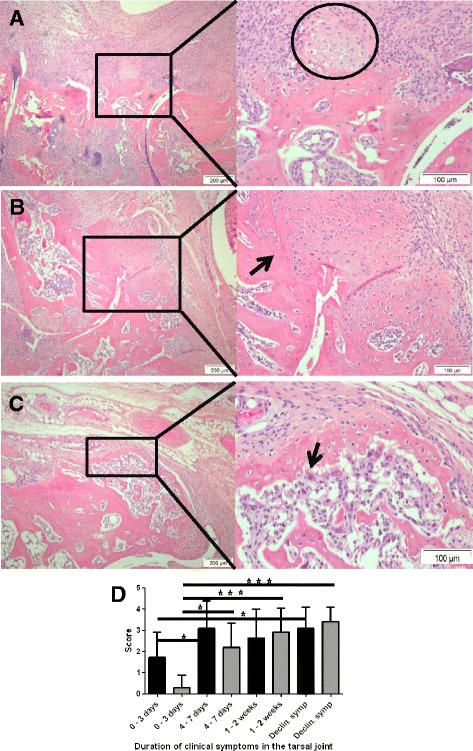
**Histopathology of tarsal joints from day 8 and until decline of symptoms. (A)** Appearance of cartilage (encircled) marks the onset of endochondral ossification (day 8). **(B)** Ankylosis has progressed from being fibrous to consist of cartilage and osteoid (day 10), with osteoid closest to the original bone (arrow). **(C)** Declining disease phase: bone formation is remodelled with OC activity observed in some animals (arrow). **(D)** Histological scores of bone erosion (black bars) and bone formation (grey bars) over time (mean +/− standard deviation (SD)). Statistics from the comparisons of individual groups is indicated by lines above the bars (Dunn’s multiple comparison test, *P <0.05, **P <0.01, ***P <0.001). OC, osteoclast.

**Week 1 to 2 after clinical disease onset:** Bone erosion (score 2.6+/−1.4) and bone formation (score 2.9+/−1.1) was pronounced and observed in 10/11 joints. Endochondral ossification was dominating (Figure 2A). Ankylosis was noted from day 8 and consisted of an inner layer of remodelled bone, followed by a cartilage layer. The outer lining layer of the bone-forming lesion consisted of mesenchymal cells (Figure 2B). In most joints, inflammation was severe, but gradually disappearing and replaced by severe fibrosis with yet active fibroblast proliferation.

**Two weeks after clinical disease onset:** The clinical severity of inflammation was declining in most joints, although 5/17 joints still showed severe clinical signs. In the joints with severe clinical arthritis, accumulation of cartilage was still apparent, while in joints with declining clinical symptoms, an increase in what was observed as mineralisation of cartilage and newly formed bone took place, in addition to modification of the newly formed bone by OCs (Figure 2C).

By comparing the development in scores for bone erosion and bone formation, respectively, a significant difference over time was observed with the greatest development in scores of bone formation (P = 0.0186 vs. *P* <0.0001) (Figure 2D).

### Gene expression in joints from mice with CIA normalises after two weeks of inflammation

**Figure 3:**
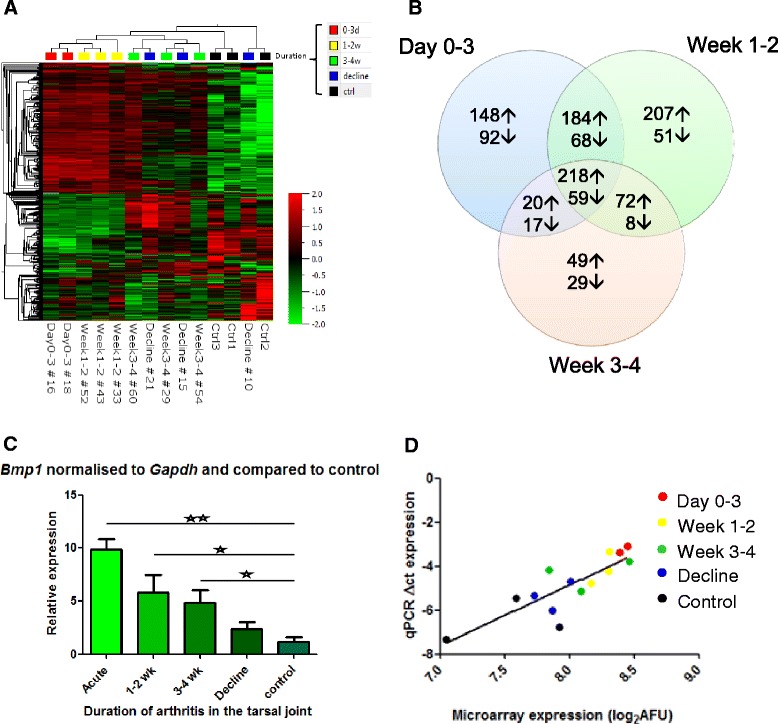
**Cluster analysis and validation of gene expression profile. (A)** Separation of samples by unsupervised hierarchical clustering. The groups are: day 0 to 3, week 1 to 2 and week 3 to 4 after onset of arthritis, and joints with declining clinical disease activity. Colours and colour intensity indicate upregulation (red) or downregulation (green) regulation of genes. **(B)** Venn diagram of the gene expression study showing the number of genes differentially expressed at the time points: day 0 to 3, week 1 to 2, week 3 to 4, and in the declining disease phase in comparison to control. **(C)** The results from the microarray study were validated with qPCR. *Bmp1* was normalised to *Gapdh* expression and compared to a control sample from non-immunised mice. Statistically significant differences between group means (δ-ct-values) are indicated with lines above the bars (Bonferroni’s multiple comparison test, *P <0.05, **P <0.01). **(D)** The gene expression level of *Bmp1* assessed by qPCR correlated significantly (r^2^ = 0.7, *P* <0.0002) with the corresponding microarray data (linear regression test).

Having examined the bone remodelling after clinical onset of arthritis histologically, we aimed at describing the time course of bone remodelling by global gene expression profiling (Figure 3A and B).

Unsupervised hierarchical clustering grouped the samples into a phase of high transcriptional activity between week 0 and 2, and, subsequently, a period of declining transcriptional activity. One sample from the declining disease phase (decline 10) clustered together with the controls, showing that the gene expression pattern normalised in the late disease phase (Figure 3A). In the first three days, 908 genes displayed a +2-fold change in the transcriptional level, while 749 genes were differentially expressed in week 1 to 2 (>2 fold change, *P* <0.05). The IPA showed that the top biological functions, canonical pathways, and upregulated genes were dominated by genes involved in arthritis and the immune system in general, in connective and skeletal tissue disorders and remodelling, as well as tissue development and function (Table S5A and B in Additional file 5).

Several potentially disease-relevant genes (*Saa3*, *Timp1*, *Postn*, *Ctsk*, *Mmp3* and *Mmp13*) demonstrated very high fold changes (between 10.6 and 45.3) (Table 1). Moreover, pregnancy–associated plasma protein A (*Pappa*) was strongly upregulated when comparing day 0 to 3 with week 1 to 2 (fold change +2.3) (Table 2). A muscle- and epidermis-specific gene expression signature was found among the most downregulated genes when comparing day 0 to 3 and control samples (Table 1).

**Table 1:** **Highly differentially expressed genes grouped according to function**

**Gene symbol**	**Ctrl (Log**_**2**_ **AFU)** ^**a**^	**Day 0 to 3** ^**b**^	**week 1 to 2** ^**c**^	**Week 3 to 4** ^**d**^	**Decline** ^**e**^	**Localisation**	**Ar vs. N** ^**f**^	**Ar vs. N** ^**g**^
*Saa3*	6.8	44.1	26.2	7.8	2.7	Plasma	18.0	2.0
*Mmp3*	7.2	23.1	11.9	5.2	2.9	Synovium	10.1	5.3
*Mmp13*	7.6	13.2	17.3	8.9	4.5	Synovium	3.5	2.3
*Timp1*	7.5	19.4	9.3	5.3	3.4	Synovium	1.9	3.5
*Ctsk*	7.8	11.0	11.7	7.6	4.8	Osteoclasts	2.8	1.7
*Atp6v0d2*	6.97	9.03	7.18	5.94	3.67	Endosome	1.5	1.0
*Postn*	8.5	10.3	9.8	5.0	2.5	Osteoblasts	4.5	1.0
*Krtdap*	7.6	0.1	0.2	4.5	14.1	Skin*	0.4	1.1
*Lce1a1/1a2*	8.2	0.1	0.1	2.8	7.9	Skin	NA	1.0
*Lor*	8.4	0.1	0.2	3.7	5.4	Skin*	0.6	1.1
*Krt1/2/5/14*	7.8	0.1	0.2	3.4	10.3	Skin*	0.8	1.0
*Mt4*	7.40	0.32	0.36	4.63	19.15	Epithelia*	0.8	1.0
*Myl2*	10.5	0.1	0.8	1.0	1.0	Muscle	0.2	0.6
*Pygm*	10.8	0.1	0.4	0.4	0.6	Muscle	0.5	0.7
*Casq1*	10.78	0.12	0.85	0.57	0.72	Muscle	0.5	0.7
*Myh4*	11.96	0.13	0.45	0.31	0.43	Muscle	0.3	0.9
*Ckm*	10.78	0.14	0.44	0.46	0.76	Muscle	0.6	0.7

^a^Mean log2 expression in non-induced control mice. Mean fold change in comparison to control: ^b^on day 0 to 3, ^c^in week 1 to 2, ^d^in week 3 to 4, ^e^in the declining disease phase. Fold change of gene expression in arthritic joints from the ^f^TNFα overexpressing model versus control [7], and the ^g^PGIA arthritis model versus control [8], *Differentially expressed epidermal specific genes in arthritic joints from the present study and the TNFα overexpressing model [7]. Ctrl, control; PGIA, proteoglycan-induced arthritis; TNFα, tumour necrosis factor alpha.

**Table 2:** **Groups of genes showing a time-dependent expression pattern**

**Gene symbol**	**Ctrl** ^**a**^	**Day 0 to 3** ^**b**^	**Week 1 to 2** ^**c**^	**Week 3 to 4** ^**d**^	**Decline** ^**e**^	
	**Log**_**2**_	**Fold change (** ***P*** **-value)**				**Protein function in relation to bone**
**Osteoclast function: highest differential expression day 0 to 3**						
***Ccl9***	6.42	***2.05 (0.01)***	1.78 (0.01)	1.61 (0.10)	1.44 (0.10)	Stimulates OC motility and polarisation through CCR1.
***Tyrobp***	10.21	***4.09 (0.03)***	3.31 (0.02)	2.47 (0.06)	1.56 (0.33)	Kinase involved in the generation of the actin cytoskeleton in OC, which is critical for bone resorption.
***Atp6v0d2***	6.97	***9.03 (0.01)***	7.18 (0.003)	5.94 (0.005)	3.67 (0.02)	ATPase proton pump involved in acidification of the extracellular resorption lacuna created by OC and early OC differentiation.
***Lif***	5.74	***2.20 (0.01)***	1.12 (0.22)	0.85 (0.39)	0.73 (0.10)	Expressed in OC and modulates the recruitment of OB to resorption surfaces in a TGFβ dependent manner.
**Early osteoblast function: highest differentially expression day 0 to 3**						
***Bmp1***	7.52	***1.86 (0.07)***	1.67 (0.10)	1.52 (0.13)	1.28 (0.30)	Metalloproteinase that may play a role on several levels of bone formation. It processes the c-terminal end of pro-collagen type I, activates other BMPs and processes BGN and DMP1. Expressed in patients with arthritis and acute fractures.
***Crtap***	6.84	***2.69 (0.02)***	2.43 (0.01)	1.66 (0.12)	1.12 (0.77)	Scaffolding protein that associates with others to hydroxylate collagen type I chains. Associated with osteogenesis imperfecta.
***Tnc***	9.19	***3.01 (0.03)***	2.67 (0.03)	2.09 (0.05)	1.72 (0.11)	Pro-inflammatory extracellular matrix protein, expressed in synovium and blood in RA patients. Involved in extracellular matrix remodelling, where it is bound by periostin.
***Lox***	7.13	***4.51 (0.11)***	3.52 (0.14)	2.49 (0.23)	1.57 (0.49)	Catalyses cross-linking of collagen type I. Is activated by BMP1.
**Late osteoblast function: highest differentially expression week 1 to 2 or week 3 to 4**						
***Sp7***	6.43	1.02 (0.90)	***1.67 (0.04)***	1.19 (0.25)	1.01 (0.95)	Transcription factor with a critical role in osteoblast differentiation.
***Spp1***	8.37	2.90 (0.05)	***4.46 (0.00003)***	3.49 (0.003)	1.98 (0.14)	OB marker, expressed in OB, OC, and osteocytes. Inhibits formation and growth of hydroxyapatite by binding the crystals. Promotes attachment of OC to bone under control of Acp5 phosphatase.
***Mgp***	10.45	2.61 (0.06)	***3.33 (0.04)***	2.45 (0.07)	1.65 (0.21)	Inhibitor of bone mineralisation.
***Sparc***	11.77	2.17 (0.04)	***2.48 (0.03)***	2.13 (0.04)	1.68 (0.07)	The protein binds hydroxyapatite when bound to collagen type I *in vitro. Sparc* null mice have normal collagen content in bone, but reduced bone formation and numbers of OC and OB.
***Enpp1***	9.08	2.72 (0.01)	***3.19 (0.0003)***	2.52 (0.01)	1.60 (0.15)	Expression is induced in OB by MDK. Essential for converting extracellular ATP to inorganic pyrophosphate, thus controlling the levels and functions in bone mineralization and soft tissue calcification. Essential for normal bone development.
***Bglap1***	9.05	0.75 (0.62)	***2.63 (0.0004)***	1.82 (0.04)	1.37 (0.13)	OB marker and serum marker of bone formation. Expressed in the late mineralisation phase. The exact role of the protein in bone is, however, debated as data is controversial.
***Ifitm5***	6.67	1.25 (0.11)	***1.47 (0.03)***	1.35 (0.05)	1.14 (0.27)	Expressed in OB and involved in mineralisation.
***Ibsp***	10.67	2.22 (0.02)	2.59 (0.01)	***2.99 (0.01)***	2.19 (0.02)	Marker of late OB differentiation. Expressed in OB, OC, hypertrophic chondrocytes and osteocytes. Binds to the AvB3 integrin and hydroxyapatite, probably mediating cell attachment to matrix. Nucleates hydroxyapatite.
**Sustained high expression week 0 to 2**						
***Col1a2***	9.94	***1.88 (0.08)***	***2.25 (0.05)***	2.03 (0.06)	1.69 (0.10)	This gene encodes the alpha2 chain of collagen type I. Collagen type I is the most abundant protein in bone and a marker of OB activity.
***Col1a1***	9.84	***1.80 (0.11)***	***2.25 (0.05)***	1.92 (0.09)	1.69 (0.14)	This gene encodes the alpha1 chain of collagen type I. Collagen type I is the most abundant protein in bone and a marker of OB activity.
***Ogn***	7.83	***2.28 (0.07)***	***3.88 (0.02)***	2.16 (0.09)	1.43 (0.34)	Proteoglycan belonging to the SLRP family. The protein is processed by BMP1 and regulates collagen type I fibrillogenesis.
***Bgn***	10.63	***2.18 (0.04)***	***2.21 (0.03)***	1.63 (0.09)	1.08 (0.72)	Proteoglycan belonging to the SLRP family. KO mice have reduced collagen synthesis, reduced bone mass and production of bone precursor cells. The protein is processed by BMP1.
***Col12a1***	8.24	***4.59 (0.04)***	***4.81 (0.04)***	2.98 (0.08)	1.75 (0.25)	Expressed by OB in areas of bone formation. COL12A1 KO mice have skeletal abnormalities with less mechanical strength and reduced matrix deposition.
***Ppib***	7.45	***2.17 (0.04)***	***2.27 (0.07)***	1.49 (0.14)	1.16 (0.64)	Associates with other proteins to hydroxylate collagen type I chains. Associated with osteogenesis imperfecta.
***Lum***	10.18	***3.37 (0.04)***	***3.97 (0.03)***	2.78 (0.05)	1.69 (0.18)	Proteoglycan belonging to the SLRP family (same as BGN).
***Hapln1***	6.72	***1.69 (0.15)***	***3.10 (0.01)***	2.88 (0.005)	2.08 (0.01)	Involved in cartilage biosynthesis by linking aggrecans and hyalyron acid. Polymorphism in this gene is associated with osteophyte formation in osteoarthritis and it is located in a susceptibility locus of AS.
***Postn***	8.52	***10.33 (0.07)***	***9.76 (0.07)***	4.97 (0.12)	2.53 (0.29)	Promotes cell survival of preosteoblasts and OB. Increases activation of pro-lysyl oxidase, which cross-links collagen type I.
***Cthrc1***	8.46	***4.78 (0.06)***	***6.79 (0.05)***	3.50 (0.10)	1.65 (0.43)	Molecule that stimulates bone formation, secreted by OC.
**Others**						
***Igf1***	9.68	1.94 (0.04)	***2.61 (0.03)***	1.82 (0.05)	1.18 (0.59)	Growth factor positively regulating bone formation. Regulates bone mineral density.
***Pappa***	5.99	***2.24 (0.0002)***	1.20 (0.06)	1.08 (0.11)	0.96 (0.68)	IGF-dependent proteinase that regulates the bioavailability of IGF. Expressed in fibroblasts, chondrocytes and OB. *Pappa* KO mice have reduced bone formation and delayed fracture-healing.
***Dmp1***	8.30	0.74 (0.42)	1.2 (0.59)	***1.81 (0.17)***	1.47 (0.30)	Extracellular matrix protein that is processed by BMP1 and involved in mineralisation of extracellular matrix. Expressed by hypertrophic chondrocytes, osteocytes, and OB.
**Tissue degradation**						
***Mmp13***	7.57	13.15 (0.02)	***17.33 (0.02)***	8.93 (0.02)	4.53 (0.05)	Bone resorption, OC- and osteoprogenitor differentiation.
***Ctsk***	7.82	11.02 (0.04)	***11.75 (0.04)***	7.58 (0.04)	4.80 (0.08)	Bone resorption. Principle enzyme degrading osteoid.
***Mmp3***	7.24	***23.11 (0.02*** *)*	11.87 (0.01)	5.24 (0.04)	2.94 (0.11)	Metalloproteinase associated with arthritis.
***Timp1***	7.53	***19.45 (0.002)***	9.33 (0.001)	5.30 (0.03)	3.44 (0.02)	Inhibitor of metalloproteinases.
***Mmp9***	7.15	***4.24 (0.02)***	3.79 (0.02)	2.95 (0.04)	2.20 (0.08)	Resorption, OC- and osteoprogenitor differentiation.
***Ctss***	8.32	***3.62 (0.04)***	2.99 (0.05)	2.29 (0.08)	1.68 (0.20)	Expressed in hypertrophic chondrocytes and OC.
***Mmp19***	5.99	***2.18 (0.02)***	2.05 (0.01)	1.45 (0.16)	1.26 (0.09)	Degradation of extracellular tissue.
***Mmp14***	7.92	2.63 (0.02)	***2.82 (0.02*** *)*	2.11 (0.02)	1.77 (0.05)	Involved in tissue destruction in RA.
***Acp5***	7.30	***3.68 (0.02)***	3.46 (0.03)	3.59 (0.03)	2.50 (0.03)	The encoded protein is a phosphatase, also called Trap. It is a marker of OC function and is extensively expressed in OC. It is activated by Cathepsin K and dephosphorylates SPP1 and IBSP. Plays an inhibitory role in collagen type I synthesis, degradation, and mineralisation.
**Genes of the BMP pathway**						
***Bmp2***	7.16	1.67 (0.30)	**1.68 (0.29)**	1.24 (0.63)	1.26 (0.59)	Ligand of the BMP pathway.
***Bmpr1a***	6.88	1.84 (0.13)	**2.01 (0.10)**	1.91 (0.11)	1.15 (0.72)	Type I receptor of the BMP pathway.
***Bmpr2***	8.36	**2.05 (0.04)**	1.65 (0.08)	1.40 (0.15)	1.30 (0.28)	Receptor type II in the BMP pathway.
***Smad2***	7.23	2.05 (0.14)	**2.14 (0.12)**	1.70 (0.22)	1.44 (0.37)	Regulatory Smad involved in BMP signalling.
***Smad6***	7.27	**0.55 (0.08)**	0.58 (0.0045)	0.72 (0.04)	0.76 (0.12)	Inhibitory Smad antagonising BMP signalling.
***Smad4***	8.16	**1.28 (0.18)**	1.27 (0.19)	1.18 (0.32)	1.04 (0.84)	Co-Smad associating with regulatory Smads.
***Smad5***	7.07	**1.40 (0.04**)	1.28 (0.06)	1.20 (0.17)	0.99 (0.92)	Regulatory Smad involved in BMP signalling.
***Bmp4***	7.68	**0.59 (0.04)**	0.68 (0.04)	0.99 (0.95)	0.95 (0.61)	Ligand of the BMP pathway.
***Bmp6***	8.44	**0.62 (0.07)**	0.75 (0.15)	0.88 (0.67)	0.93 (0.63)	Ligand of the BMP pathway.
***Bmp7***	5.82	**0.78 (0.19)**	0.86 (0.31)	0.94 (0.67)	1.26 (0.17)	Ligand of the BMP pathway.
***Inhba***	7.20	**5.65 (0.01)**	3.94 (0.01)	2.09 (0.07)	1.17 (0.77)	Non-steroidal hormone reported to increase bone formation in transgenic mice overexpressing human inhibin A. Associated with human arthritis.
***Acvr2a***	7.23	1.27 (0.33)	***1.29 (0.03)***	1.18 (0.04)	0.91 (0.34)	Type II receptor of the TGFβ pathway.
***Acvr2b***	6.21	***0.80 (0.01)***	0.73 (0.0034)	0.76 (0.03)	0.85 (0.29)	Type II receptor of the TGFβ pathway.
**Genes of the WNT pathway**						
***Fzd1***	6.91	***1.70 (0.05)***	1.57 (0.05)	1.39 (0.08)	1.18 (0.37)	Receptor of the WNT pathway.
***Hif1a***	8.35	***4.23 (0.02)***	3.56 (0.02)	2.49 (0.03)	1.43 (0.34)	Regulates the cellular response to hypoxia during endochondral bone formation and inhibits WNT signalling in OB in cooperation with Sp7.
***Sost***	7.28	***0.48 (0.03)***	0.61 (0.02)	0.62 (0.03)	0.73 (0.15)	WNT antagonist and marker of mature osteocytes.
***Ndrg2***	10.56	***0.18 (0.01)***	0.31 (0.0014)	0.37 (0.01)	0.52 (0.01)	Down-regulates WNT-signalling mediated TCF-promotor activity and GSK3β phosphorylation synergistically with PRA1.
***Dkk1***	7.19	***0.67 (0.18)***	0.85 (0.25)	1.15 (0.32)	1.09 (0.43)	Antagonist of the WNT pathway.
***Tcf4***	7.96	1.46 (0.09)	***1.73 (0.03)***	1.30 (0.28)	0.99 (0.94)	Transcription factor of the WNT pathway.

^a^Mean log2 expression in non-induced control mice. Mean fold change and p-value in comparison to control: ^b^on day 0 to 3, ^c^in week 1 to 2, ^d^in week 3 to 4, ^e^in the declining disease phase. The highest differential expression value and corresponding p-value of each gene are shown in bold italics. AS, ankylosing spondylitis; BMP, bone morphogenetic protein; BGN, biglycan; CCR1, C-C chemokine receptor type 1; Ctrl, control; DMP1, dentin matrix acidic phosphoprotein1; IGF, insulin-like growth factor; KO, knockout; MDK, midkine; OB, osteoblast; OC, osteoclast; RA, rheumatoid arthritis; SLRP, leucine rich repeat containing 6; TCF, T-cell-specific transcription factor; TGFβ, transforming growth factor beta.

In week 3 to 4 after onset of disease, the number of two-fold differentially expressed genes had decreased to 353 (Figure 3B). The pathway profile changed accordingly, and was dominated by tissue remodelling and developmental pathways, although an inflammatory gene expression signature was still present (Table S5C and D in Additional file 5).

A comparison of gene expression between week 0 to 2 and week 3 to 4 revealed considerable up-regulation of several epidermal genes (fold changes between 3.8 and 13.9) in the samples collected three to four weeks after onset of arthritis (Table 1).

The expression pattern of 15 differentially expressed genes was verified by qPCR. Of these, 12 genes correlated significantly with the gene expression pattern obtained from the microarray profile, while three genes showed the same expression pattern, but the correlation was not significant. The expression of *Bmp1* is shown in Figure 3C and D (r^2^ = 0.7, p < 0.0002), and the remaining validation data are shown in Figure S6 in Additional file 6 and Figure S7 in Additional file 7 and with the corresponding r^2^ and *P*-values in Table S8 in Additional file 8.

To identify genes associated with the observed bone pathology, we focused on genes, differentially expressed at the different time-points, which were related to bone and skeleton in the IPA Bio Functions. The genes and associated Bio Functions reflected the bone remodelling process (Table S9 in Additional file 9), and their number and effect size declined over time. The genes and their expression profiles are shown individually in Table S10 in Additional file 10.

### Distinctive expression profiles of genes involved in osteoblast and osteoclast functions

**Figure 4:**
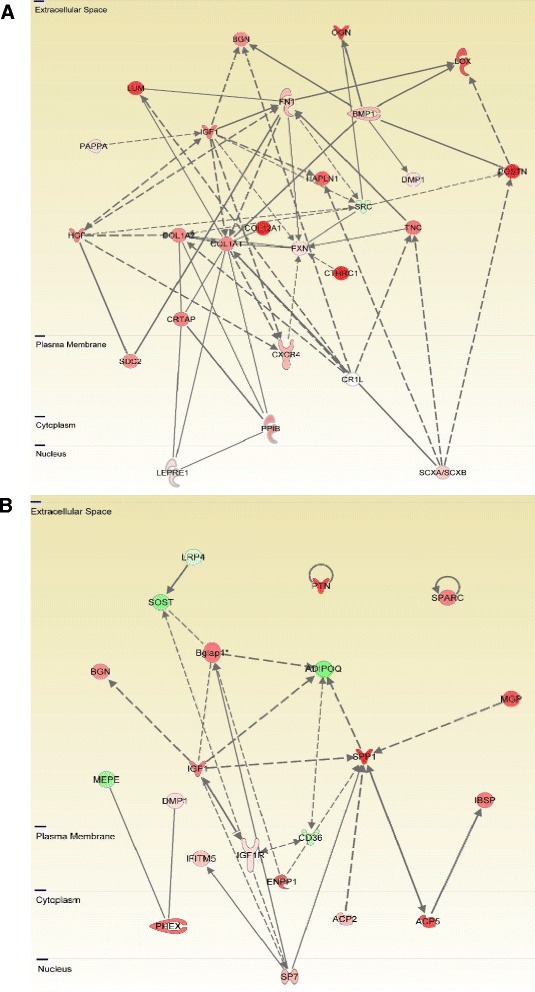
**Gene expression networks during osteoblast activity in early and late phases of joint inflammation. A)** A network of genes related to osteoblast activity, in particular collagen type I assembly and cross-linking, during the early phase of clinical disease. Colours and colour intensity indicate upregulation (red) or downregulation (green) regulation of genes at day 0 to 3, where most of the genes showed the greatest fold change in comparison to non-induced controls. One exception is *Dmp1*, which became upregulated at week 3 to 4. **B)** A network of genes related to osteoblast activity, in particular mineralisation, during the late phase of clinical disease. Colours and colour intensity indicate upregulation (red) or downregulation (green) regulation of genes at week 1 to 2, where most of the genes showed the greatest fold change in comparison to non-induced controls. One exception is *Ibsp*, which was most highly upregulated in week 3 to 4.

The differentially expressed genes, related to bone, from Table S10 in Additional file 10, were divided into profiles and are shown separately in Table 2. Profile one encompassed genes with the highest expression on day 0 to 3 with a subsequently declining profile. These were in particular genes encoding proteins involved in OC differentiation and function (*Ccl9, Tyrobp, Atp6v0d2, Lif*; fold changes between 2.0 and 9.2), in addition to ECM formation and collagen type 1 assembly (*Bmp1, Crtap, Tnc, Lox*; fold changes between 1.9 and 10.6) [9-14] (Table 2, Figure 4A, and Table S10 in Additional file 10). In profile two, genes with the highest expression in week 1 to 2, followed by a declining expression pattern, were included. These were in particular genes encoding proteins involved in mineralisation and osteoblast function (*Sp7*, *Spp1, Mgp, Sparc, Enpp1, Bglap1, Ifitm5*; fold changes between 1.5 and 4.5) [15-21] (Table 2, Figure 4B and Table S10 in Additional file 10). Integrin-binding sialoprotein (*Ibsp*; fold change +3.0) also belongs to this group [20], but showed the highest expression in week 3 to 4. Moreover, a large group of genes showed a sustained differential expression pattern at day 0 to 3 and week 1 to 2. These included genes involved in osteoblast functions and ECM formation (*Col1a1, Col1a2, Ogn, Bgn, Col12a1, Ppib, Lum, Hapln1, Cthrc1*, *Postn*; fold changes between 2.2 and 10.3) [13,14,22-27] (Table 2). Finally, tissue destruction was reflected by up-regulation of the genes for four metalloproteinases (MMPs): the MMP inhibitor *Timp1;* acid phosphatase 5 (*Acp5*); cathepsin K [28]; cathepsin S [29], and additional cathepsins. These genes were among the most highly expressed, throughout disease development (Table 1, Table 2 and Table S10 in Additional file 10).

### Differential expression of genes belonging to the BMP pathway

**Figure 5:**
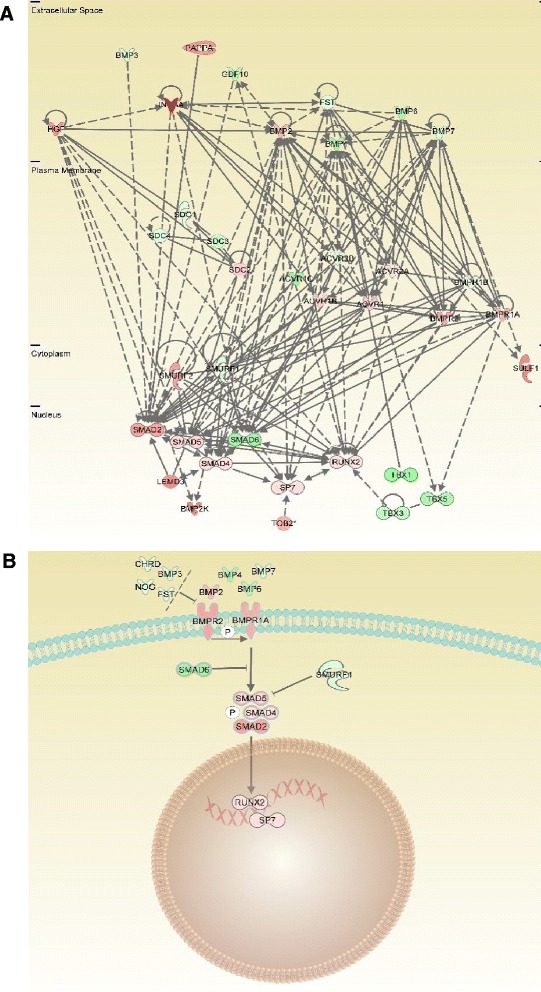
**Differentially expressed genes related to BMP signalling at day 0 to 3 of arthritis in comparison to non-induced controls.** Regulation of genes is indicated by colour and colour intensity (red = upregulated, green = downregulated). **(A)** Network of BMP related genes in general. **(B)** Functional pathway of BMP signalling. BMP, bone morphogenetic protein.

Differentially expressed genes related to the BMP pathway included the genes encoding the BMP type I and type II receptors (*Bmpr1a* and *Bmpr2*, fold change +2 at week 1 to 2 and day 0 to 3, respectively), and proteins downstream of the BMP signalling pathway (*Smad2* and *Smad6*, fold change +2.1 at week 1 to 2 and −1.8 at day 0 to 3, respectively) (Table 2, Figure 5 and Table S10 in Additional file 10). The BMP ligand *Bmp2* (fold change +1.7 at day 0 to 3) together with *Smad4* (fold change +1.3 at day 0 to 3) and *Smad5* (fold change +1.4 at day 0 to 3) showed the same marginal change in expression pattern. The BMP ligands *Bmp4*, *Bmp6,* and *Bmp7* were slightly downregulated (fold change between −1.7 to −1.3 at day 0 to 3).

High upregulation of the gene for Inhibin beta A (*Inhba*; fold change +5.6 at day 0 to 3) was found, in addition to minor expression changes in the genes of some of the transforming growth factor beta (TGFβ) receptors (*Acvr2a* and *Acvr2b)* bound by this protein (Table 2 and Figure 5A).

### Differential expression of genes belonging to the WNT pathway

Within the group of genes related to the WNT pathway, the genes for the receptor FZD1 (fold change +1.7 at day 0 to 3) and transcription factor TCF4 (fold change +1.7 at week 1 to 2) were differentially expressed, in addition to a number of WNT inhibitors. These included the hypoxia-induced *Hif1a*, which showed more than 4-fold up-regulation and *Sost* and *Ndrg2*, which were between 2 and 5.6-fold downregulated. The well-described WNT inhibitor *Dkk1* was slightly downregulated (Table 2 and Table S10 in Additional file 10).

### Comparison to genome-wide association studies in human arthritis

Some of the genes differentially expressed in the herein presented study have been linked to human arthritis in genome-wide association studies (GWAS) (Table 2). Of those genes, several are involved in cartilage- and bone remodelling (underlined in Table 3).

**Table 3:** **Differential expression of genes associated with human arthritis in GWAS studies**

**Gene symbol**	**Ctrl** ^**a**^	**0 to 3d vs. Ctrl** ^**b**^	**1 to 2w vs. Ctrl** ^**c**^	**3 to 4w vs. Ctrl** ^**d**^	**Decline vs. Ctrl** ^**e**^	**Protein function**
	**log2**	**Fold change (** ***P*** **-value)**				
**Ankylosing spondylitis**						
***Hapln1***	**6.72**	**1.69 (0.15)**	**3.10 (0.01)**	**2.89 (0.005)**	**2.08 (0.01)**	**Involved in cartilage development and bone formation. Associated with osteophyte formation in OA [** 30 **].**
***Edil3***	**5.58**	**1.53 (0.14)**	**2.95 (0.003)**	**3.07 (0.005)**	**2.33 (0.11)**	**Integrin ligand with a proposed role in cartilage development and angiogenesis. Inhibitor of the WNT pathway [** 30 **].**
***Ano6***	**7.22**	**2.25 (0.05)**	**1.88 (0.04)**	**1.61 (0.14)**	**1.19 (0.60)**	**Regulates phosphatidylserine exposure on the cell surface, which is essential for mineralisation of bone and involved in osteoclast formation [** 30 **,** 31 **].**
*Psmg1*	6.95	2.04 (0.002)	1.91 (0.002)	1.37 (0.03)	1.03 (0.78)	Proteasome Assembly Chaperone 1. The gene is, in addition, associated with inflammatory bowel disease [32].
**Rheumatoid arthritis**						
*Anapc4*	6.85	1.77 (0.0008)	1.91 (0.0003)	1.45 (0.02)	1.18 (0.45)	An SNP close to this gene was associated with RA susceptibility [33]. The encoded protein is an E3 ubiquitin ligase involved in control of the cell cycle, muscle cell function, and function of neurons [34].
*Cd84*	5.61	3.07 (0.01)	3.01 (0.004)	2.17 (0.02)	1.51 (0.12)	Membrane protein involved in immune function. High expression of the gene was associated with a positive response to Etanercept treatment [35].
***Il6st***	**9.44**	**2.41 (0.03)**	**1.79 (0.06)**	**1.52 (0.10)**	**1.09 (0.76)**	**Signal transducing subunit for cytokines belonging to the IL-6 family. Activates Stat-3, which plays a role in OC** **and OB** **differentiation and their interaction [** 36 **].**
*Ptpn2*	6.68	1.74 (0.06)	1.78 (0.02)	1.32 (0.30)	1.46 (0.16)	Protein phosphatase involved in T-cell activation by regulation of the JAK/STAT signalling pathway [33,37,38].
*Cdk6*	5.88	2.51 (0.10)	2.35 (0.08)	2.11 (0.08)	1.64 (0.21)	Cyclin-dependent kinase involved in proliferation of lymphocytes [39].
*Prkcq*	7.42	0.48 (0.17)	0.65 (0.26)	0.55 (0.16)	0.61 (0.32)	Kinase involved in activation of NFκB and AP-1 transcription factors [39].
*Prkch*	7.20	1.36 (0.04)	1.43 (0.07)	1.58 (0.06)	0.99 (0.95)	Serine/threonine kinase. The association with RA is suggestive [38].
**Osteoarthritis**						
*Gnl3*	7.81	2.41 (0.11)	2.10 (0.08)	2.17 (0.07)	1.47 (0.34)	Nucleostemin. Increased in *in vitro* cultures of chondrocytes from patients with OA [40].
*Glt8d*	7.18	1.97 (0.03)	1.99 (0.02)	1.43 (0.05)	1.13 (0.71)	Involved in glycosylation of cartilage proteins [40].
*Pla2g*	5.96	1.47 (0.16)	1.67 (0.04)	2.06 (0.03)	1.71 (0.04)	Cytosolic phospholipase A2 enzyme. mRNA transcript was abundantly expressed in chondrocytes from OA patients [41].
*Ptgs2*	6.91	2.66 (0.01)	1.72 (0.07)	1.15 (0.41)	1.56 (0.03)	Cyclooxygenase 2 (COX-2). mRNA transcript was abundantly expressed in chondrocytes from OA patients [41].
***Col12a1***	**8.24**	**4.56 (0.04)**	**4.82 (0.01)**	**2.97 (0.04)**	**1.74 (0.22)**	**A SNP close to this gene has been associated with OA susceptibility [** 40 **]. The encoded protein is expressed by OB in areas of bone formation. KO** **mice have skeletal abnormalities with less mechanical strength and reduced matrix deposition [** 24 **].**

^a^Mean log2 expression in non-induced control mice. Mean fold change and *P*-value in comparison to control: ^b^on day 0 to 3, ^c^in week 1 to 2, ^d^in week 3 to 4, ^e^in the declining disease phase. Genes shown in bold are encoding proteins with a known function in cartilage- or bone remodelling. Ctrl, control; GWAS, genome-wide association studies; IL-6, interleukin 6; KO, knockout; NFκB, nuclear factor kappa B; OA, osteoarthritis; OB, osteoblast; OC, osteoclast; RA, rheumatoid arthritis; WNT, wingless-type.

## Discussion

In this study, we have shown that in CIA, major histopathological changes, comprising inflammation as well as bone formation and ankylosis, occur within the first two weeks after the inflammatory symptoms become clinically apparent in a joint. Moreover, we have shown that the histopathology is reflected at the gene expression level, where a number of genes, coding for proteins important in inflammation and bone remodelling, are differentially expressed within the same time period after disease onset. This suggests that the CIA model is relevant for the osteoproliferative inflammatory arthritis in PsA and AS.

Bone formation in the murine CIA model has been described as starting in the subacute inflammatory disease phase, two to four weeks after onset of arthritis, but mainly playing a role in the healing state two to three months after onset [2]. However, no studies disclose any molecular details regarding bone formation throughout the arthritis disease course. Recently, Schett *et al*. [3] performed a histological study of new bone formation in the rat CIA and AIA models. In their study, periosteal proliferation was noted on day 3 (CIA) and day 5 (AIA) after onset of arthritis and new bone formation peaked at day 27 after onset of arthritis in both models.

The link between joint inflammation and bone remodelling is not clear. In human arthritis, areas with inflammation were shown to be predictive of bone formation [42], but bone formation does not seem to be blocked by anti-tumor necrosis factor alpha (TNFα) treatment [43]. In the present study, we have shown that in the murine CIA model, bone formation is (1) initiated during active inflammation, (2) develops in parallel with the inflammation, and (3) that the bone mineralises and remodels when clinical inflammatory signs are declining.

The molecular events underlying inflammation and bone remodelling were investigated using gene expression profiling. The results are based on a first kinetic mapping of global gene expression in the joint during clinical onset and development of arthritis. Expression of specific genes might differ depending on the microanatomical site within the joint and future studies of proteins important in the interplay between inflammation and bone formation should address this. We found that the bone pathology observed histologically was reflected in the Bio Functions, significantly associated with differentially expressed genes, from onset to decline of clinical arthritis in the joint. Moreover, genes involved in inflammation and bone remodelling were co-expressed during the first two weeks after onset of clinical arthritis and, hereafter, the gene expression gradually normalized to control levels, while the clinical inflammation was declining. A muscle-specific signature was observed among the most downregulated genes during the first two weeks after onset of arthritis. This may reflect muscle inactivity and atrophy observed in the model [44] and may translate to the impaired muscle function observed in arthritis patients. However, since animals in the acute disease phase also lose weight, this may partly explain some of the gene expression changes observed in muscle-specific genes.

Several metalloproteinases and cathepsins associated with human arthritis showed sustained, high expression during the entire disease course (Table 1, and Table S10 in Additional file 10). These reflect tissue destruction, in addition to being a link between bone erosion and bone formation. MMP9 and MMP13 have been shown to play a crucial role in endochondral bone formation, as well as in matrix degradation and in OC and osteoprogenitor differentiation [45,46].

**Figure 6:**
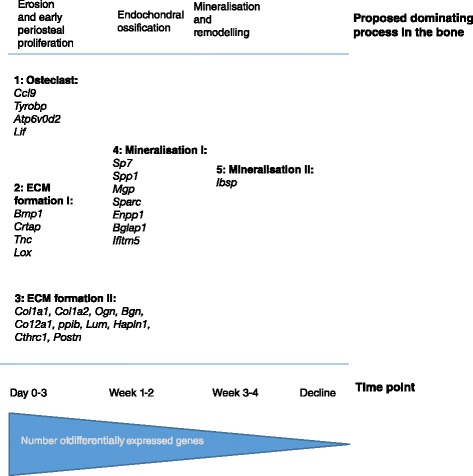
**Bone remodelling in the CIA model.** Model of bone remodelling during the course of clinical arthritis. The proposed dominating process in the bone is shown in the top part of the figure. Differentially expressed genes, encoding proteins with a function in bone remodelling, are divided into groups according to function and time point of highest differential expression in comparison to the control. All genes are upregulated in comparison to the control at the indicated time-point. Group 1: genes encoding proteins involved in osteoclast function and differentiation; Group 2: genes encoding proteins with a function in extracellular matrix formation and collagen type I assembly (highest differential expression at day 0 to 4); Group 3: genes encoding proteins with a function in extracellular matrix formation and collagen type I assembly (sustained high differential expression on day 0 to 4 and week 1 to 2); Group 4: genes with a function in mineralisation of bone (highest differential expression in week 1 to 2); Group 5: genes with a function in mineralisation of bone (highest differential expression during week 3 to 4). CIA, collagen-induced arthritis; ECM, extracellular matrix.

Genes with the highest fold change at day 0 to 3 after onset of clinical arthritis reflected osteoclast function and differentiation, in addition to ECM formation and collagen type 1 assembly, while genes showing the highest fold change at week 1 to 2 or week 3 to 4 encompassed a well-known group of OB markers and genes involved in mineralisation (Table 2, Figure 4A and B). These variations in gene expression pattern mirror the development in OB differentiation and function, as gene expression is specific for the differential stages of the OB [47]. The gene expression profiles followed the histological observations: bone erosion, periosteal proliferation and osteoid secretion, was observed in the initial phase followed by onset of endochondral bone formation and mineralisation in weeks 1 to 2. The data support a model for bone remodelling during arthritis, where bone erosion is mainly taking place during the very early phase of clinical arthritis, with the onset of tissue remodelling and bone formation following shortly after, developing in parallel with the inflammation. When the inflammatory response ceases, the addition of new bone decreases, and the hitherto created bone is remodelled and mineralised. As suggested by the histological results, the newly formed bone may be created in different processes depending on the microanatomical site. Thus, addition of bone in the periosteum takes place first, without preceding cartilage formation, while osteophytes at the joint margin are created in a process resembling endochondral ossification (Figure 6). A comparison of the present data to the proteoglycan-induced arthritis (PGIA) model reveals a high degree of similarity concerning the mixed anabolic and catabolic bone remodelling profile [8]. In contrast, the TNFα-transgenic arthritis mouse model showed a primarily catabolic bone remodelling profile, while genes representing the osteoproliferative response showed no or marginally differential expression [7]. This is in line with a general comprehension of the TNFα-transgenic mouse as a mainly erosive model, while the PGIA and CIA models demonstrate osteoproliferative arthritis. All three models showed a strong up-regulation of *Mmps* and *Timp1*. Data are compared in Table S10 Additional file 10.

Highly differentially expressed genes encoding proteins involved in bone formation between day 0 to 3 of clinical arthritis are potential links between inflammation and bone formation. Two of these genes are the insulin-like growth factor (IGF)-dependent proteinase pregnancy-associated plasma protein A gene (*Pappa*) and periostin (*Postn*). In addition, genes particularly involved in early ECM formation, preceding bone formation, are potential candidates. The PAPP-A protein is an anabolic growth factor in bone *in vivo* and *in vitro* [48,49] and regulates bioavailability of Insulin-like growth factor (IGF) involved in bone formation [50], and also differentially expressed. Expression of *Pappa* is stimulated by inflammatory cytokines in several cell types, including OBs, and macrophages isolated from artherosclerotic lesions [51,52]. It has recently been identified as a marker of inflammation in acute coronary syndrome [53]. Furthermore, *Pappa* knockout mice display disturbed bone formation [54,55], while mice overexpressing *Pappa* have increased bone formation [48,49]. Thus, *Pappa* expression might be induced in the early inflammatory environment and the protein could be involved in initiating bone formation in the CIA model.

The *Postn* gene is one of the highly differentially expressed genes with a proposed role in bone remodelling. It is preferentially located in the periostium and is re-expressed during fracture repair, mechanical stress, and in RA [14]. The expression is regulated by BMP2 and TGFβ. Overexpression of periostin increases bone formation possibly by promoting cell survival of preosteoblasts and OBs [56]. By binding fibronectin, tenascin and BMP1, it increases the activation of pro-lysyl oxidase, which in turn cross-links collagen [14]. Moreover, BMP1 has been linked to arthritis and bone formation [57,58] and may increase bone formation in several ways.

In weeks 1 to 2 after clinical onset, many genes reflecting the mineralisation of osteoid showed the highest differential expression over time in comparison to control (Table 2 and Figure 4B). Integrin-binding sialoprotein (*Ibsp*) is involved in the formation of crystals, as well as cell attachment to hydroxyapatite [20]. Osteonectin (*Sparc*) binds and connects hydroxyapatite to collagen type I [21], while osteonectin (*Spp1*) binds hydroxyapatite crystals, inhibiting their formation [21]. Moreover, these genes also reflect the complexity of interactions between ECM, OCs and OBs, as many of them have dual functions in bone remodelling.

In summary, the network of regulated genes involved in collagen type I assembly represented in Figure 4A, might play an important role in arthritis-associated ECM formation, leading to fibrosis and osteophyte formation.

Of particular interest in arthritis-associated bone remodelling are molecules belonging to the BMP and WNT signalling pathways. In human arthritis, the BMP ligands 2 and 6 [59] and their receptors BMPR1a and BMPR2 [60] were upregulated, while BMP ligands 4 and 5 were downregulated in RA and osteoarthritis (OA) synovium in comparison to normal control samples. Our results showed a similar up-regulation of the genes for the receptors BMPR1a and BMPR2, but down-regulation of *Bmp4*, *Bmp6* and *Bmp7* (Figure 5).

One of the strongly upregulated genes in this study is *Inhba* (Inhibin beta A), which may stimulate the TGFβ signalling pathway through binding to receptors Acv1b, Avr2a and Avr2b. This non-steroidal hormone regulating follicle-stimulating hormone (FSH) secretion is reported to increase bone formation in a knock-in transgenic mouse [61], and has been associated with human arthritis [62].

A profile of WNT signalling is not distinct in our gene expression data, since most of the genes belonging to this pathway were marginally differentially expressed compared to the control samples. However, a number of WNT inhibitors were differentially expressed. These included the upregulated *Hif1a*. The encoded protein is a hypoxia inducible factor that plays a role in angiogenesis during endochondral bone formation. Together with Sp7, it inhibits the WNT signalling pathway [63]. Moreover, WNT inhibitors *Sost* and *Ndgr2* were both downregulated and *Dkk1*, the master regulator of WNT signalling, was slightly downregulated. This could be expected during anabolic bone conditions. *Sost* and *Dkk1* downregulation has been associated with proteoglycan-induced arthritis in mice, and DKK1 with human AS [8].

In Table 3, several genes identified in GWAS of human arthritis are listed together with the expression data from the CIA model found in the present study. The five underlined genes encode proteins with a function in cartilage or bone remodelling, and are potential therapeutic candidate genes. The expression of those genes highlights the importance of the CIA model for studies of osteoproliferative inflammatory arthritic diseases.

## Conclusions

Bone remodelling in the CIA model results in ankylosis within one to two weeks after clinical disease onset and occurs simultaneously with the local inflammatory process in the joint. Furthermore, in this study, we have identified networks of genes involved in early and late bone formation, as well as genes associated with human arthritis, which may provide potential targets for modulating bone formation in arthritis.

Taken together, this suggests that the CIA model is an important tool in investigations of the relationship between inflammation and bone formation in the search for therapeutic targets to prevent ankylosis in inflammatory arthritis.

## Additional files

Additional file 1: Table S2. A and B: Overview of samples used for histology and global gene expression study. Additional file 2: Figure S1. Illustration of histological bone scores. **(A)** Bone erosion and formation score 0: no pathological changes. **(B)** Bone erosion score 2. **(C)** Bone formation score 2. **(D)** Bone erosion score 4. **(E)** Bone formation score 4. Additional file 3:
**RMA gene default summary of array data for review.**
Additional file 4: Table S4. Table of assay IDs for specific primer/probe sets (Life Techologies) used for validation of gene expression at AROS Applied Biotechnology A/S. Additional file 5: Table S5.
**(A)** Comparison of samples from day 0 to 3 versus control. **(B)** Comparison of samples from week 1 to 2 versus control. **C)** Comparison of samples from week 3 to 4 versus control. **(D)** Comparison of samples from the declining disease phase versus control. Additional file 6: Figure S6. Validation of selected genes by qPCR showing correlation between the microarray data (X-axis, log2-transformed values) and the qPCR data (Y-axis, δCt values). δC_t,gene_ is calculated as C_t,Gapdh_-C_t,gene._ Color coding, Red: day 0 to 3; Yellow: week 1 to 2; Green: week 3 to 4; Blue: declining disease phase; Black: control. Additional file 7: Figure S7. Validation of selected genes by qPCR showing a non-significant correlation between the microarray data (X-axis, log2 transformed values) and the qPCR data (Y-axis, δCt values). δC_t,gene_ is calculated as C_t,Gapdh_-C_t,gene._ Color coding, Red: day 0 to 3; Yellow: week 1 to 2; Green: week 3 to 4; Blue: declining disease phase; Black: control. Additional file 8: Table S8. Correlation data and *P*-values for correlation between microarray and qPCR data. Additional file 9: Table S9. Selected Bio Functions related to bone. The Bio Functions that form the basis of the list of genes encoding proteins with a function in bone remodelling. Additional file 10: Table S10. List of genes encoding proteins with a suggested function in bone remodelling. The list was created from data in the selected Bio Functions and supplemented with genes based on literature search. The data are also compared to data from other gene expression studies. A list of references is available (please contact the corresponding author).

## Abbreviations

AIA: antibody-induced arthritis
AS: ankylosing spondylitis
BMP: bone morphogenetic protein
CFA: complete Freund’s adjuvant
CIA: collagen-induced arthritis
CII: collagen type II
ECM: extracellular matrix
ERK: extracellular signal-regulated kinases
FSH: follicle-stimulating hormone
GWAS: genome-wide association studies
IFA: incomplete Freund’s adjuvant
IGF: insulin-like growth factor
IPA: Ingenuity Pathway Analysis
MMP: matrix metallopeptidase
OA: osteoarthritis
OB: osteoblast
OC: osteoclast
PGIA: proteoglycan-induced arthritis
PsA: psoriatic arthritis
RA: rheumatoid arthritis
TGFβ: transforming growth factor beta
TNFα: tumor necrosis factor alpha
WNT: wingless-type
