# Supplementary material for: Kinetics of gene expression and bone remodelling in the clinical phase of collagen-induced arthritis
Source: Arthritis Res Ther. 2015 Mar 5;17(1):43. doi: 10.1186/s13075-015-0531-7 (PMC4391727; doi:10.1186/s13075-015-0531-7)
Supplement: Additional file 1: Table S2. — A and B: Overview of samples used for histology and global gene expression study. [file 13075_2015_531_MOESM1_ESM.pdf]

Additional table 2A and B

Overview of samples used for histology and global gene expression study

Additional table 2A: Joints used for the histological study

Duration of clinical inflammation	0-3 days	4-7 days	1-2 weeks	>2 weeks	> 2 weeks, declined inflammation
Number of joints	14	10	11	5	12
Clinical score at sampling	3	3	3	3	<2*

*The joints had had a clinical score of 3 for minimum 2 weeks. Hereafter, the clinical score had declined minimum 1 score.

Additional table 2B: Joints used for the global gene expression study

Duration of inflammation	0-4 days	1-2 weeks	3-4 weeks	>2 weeks, declined inflammation
Number of joints	3	3	3	3
Clinical score at sampling	3	3	3	<2*

* The joints had had a clinical score of 3 for minimum 2 weeks. Hereafter, the clinical score had declined minimum 1 score.
