# Supplementary figures and images for: Kinetics of gene expression and bone remodelling in the clinical phase of collagen-induced arthritis
Source: Arthritis Res Ther. 2015 Mar 5;17(1):43. doi: 10.1186/s13075-015-0531-7 (PMC4391727; doi:10.1186/s13075-015-0531-7)

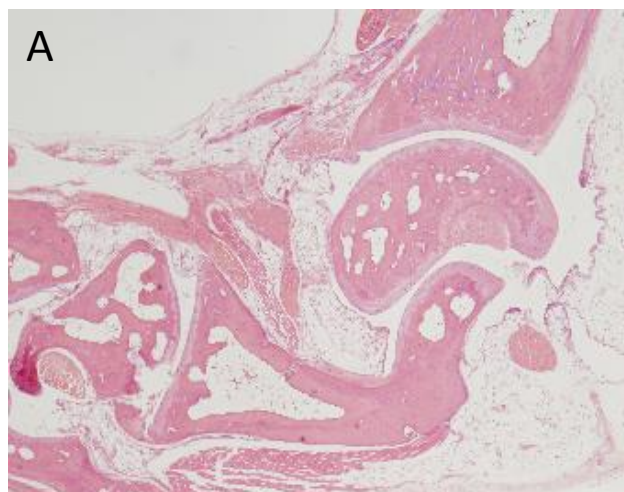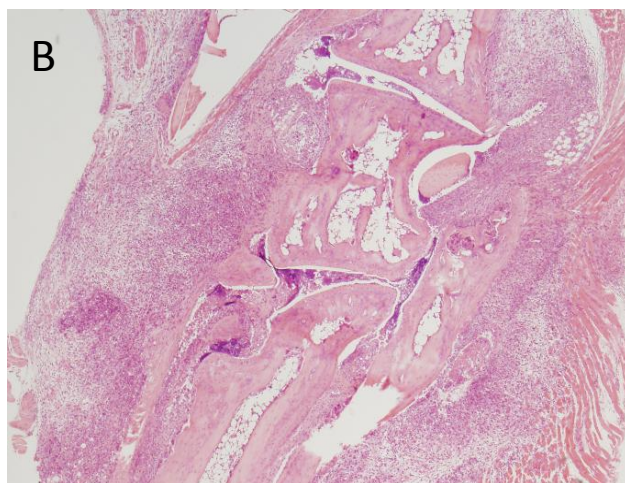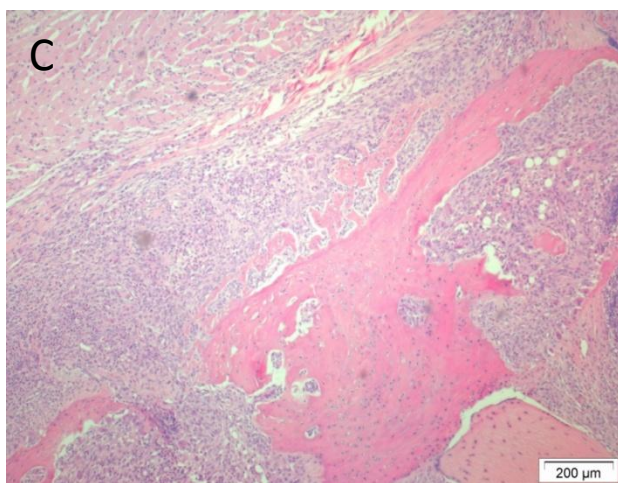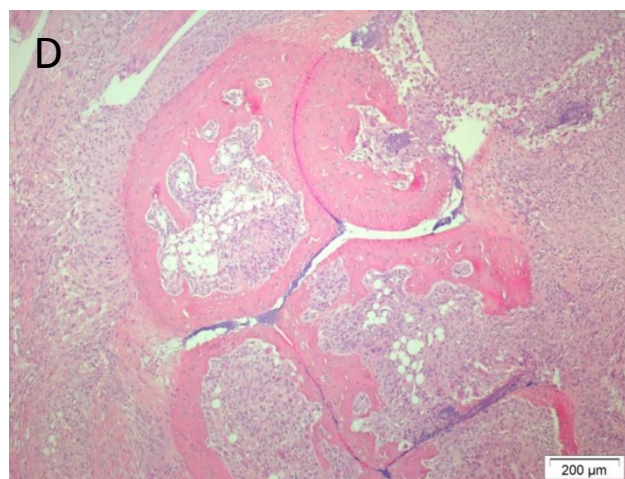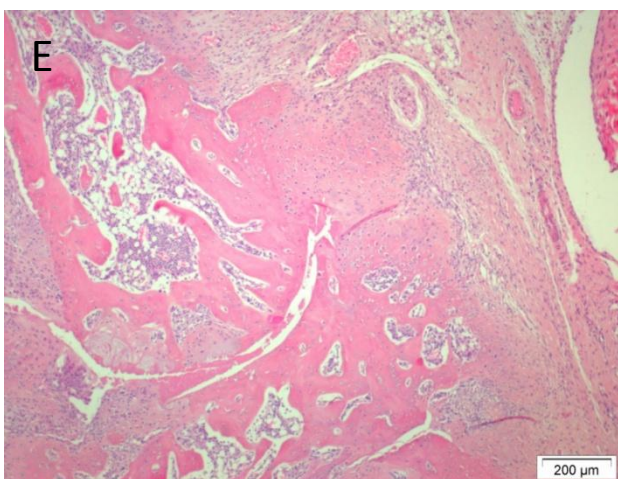

Supplement: Additional file 2: Figure S1. — Illustration of histological bone scores. (A) Bone erosion and formation score 0: no pathological changes. (B) Bone erosion score 2. (C) Bone formation score 2. (D) Bone erosion score 4. (E) Bone formation score 4. [file 13075_2015_531_MOESM2_ESM.pdf]

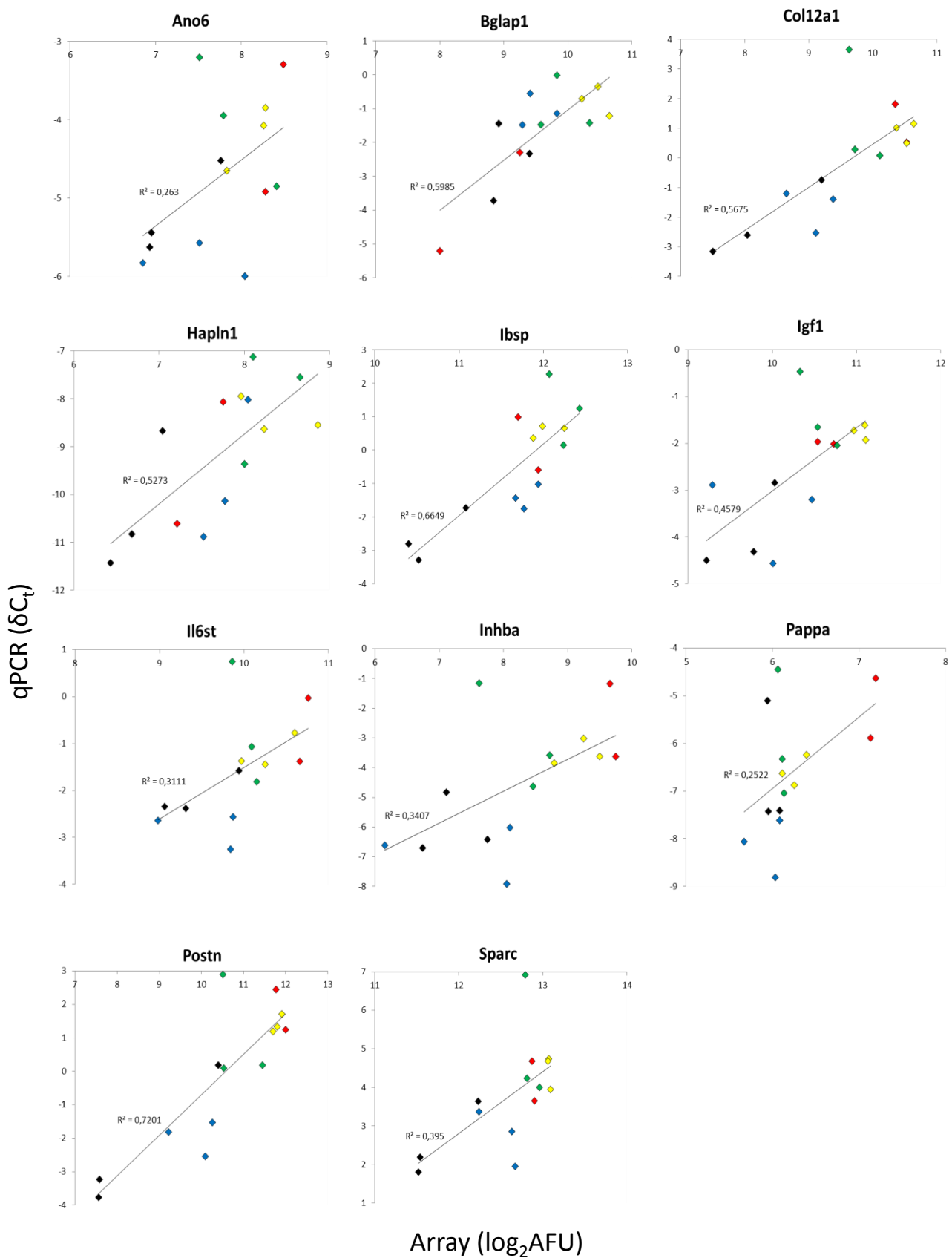

Supplement: Additional file 6: Figure S6. — Validation of selected genes by qPCR showing correlation between the microarray data (X-axis, log2-transformed values) and the qPCR data (Y-axis, δCt values). δCt,gene is calculated as Ct,Gapdh-Ct,gene. Color coding, Red: day 0 to 3; Yellow: week 1 to 2; Green: week 3 to 4; Blue: declining disease phase; Black: control. [file 13075_2015_531_MOESM6_ESM.pdf]

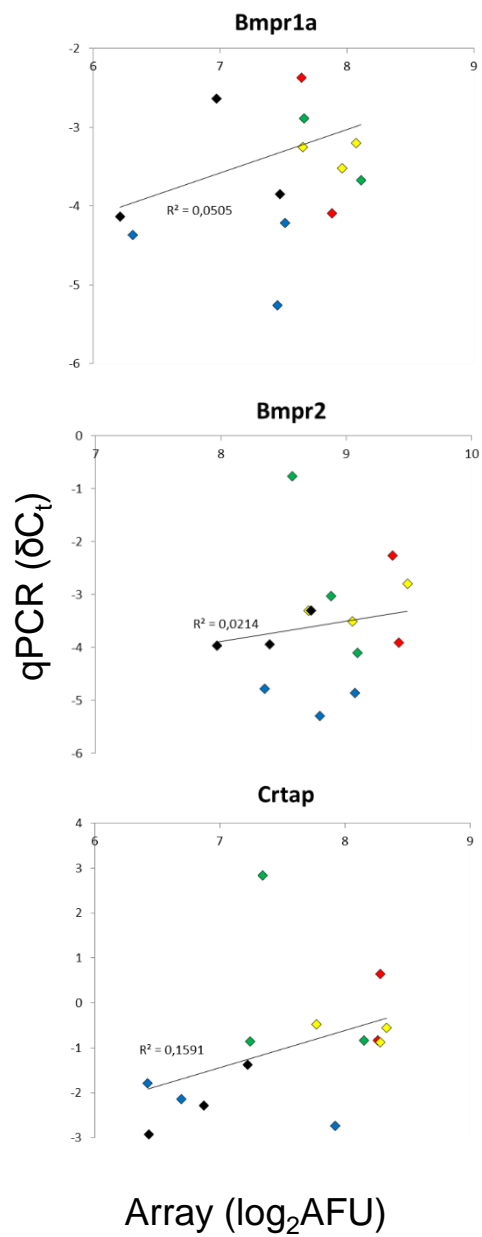

Supplement: Additional file 7: Figure S7. — Validation of selected genes by qPCR showing a non-significant correlation between the microarray data (X-axis, log2 transformed values) and the qPCR data (Y-axis, δCt values). δCt,gene is calculated as Ct,Gapdh-Ct,gene. Color coding, Red: day 0 to 3; Yellow: week 1 to 2; Green: week 3 to 4; Blue: declining disease phase; Black: control. [file 13075_2015_531_MOESM7_ESM.pdf]
