# Supplementary material for: Kinetics of gene expression and bone remodelling in the clinical phase of collagen-induced arthritis
Source: Arthritis Res Ther. 2015 Mar 5;17(1):43. doi: 10.1186/s13075-015-0531-7 (PMC4391727; doi:10.1186/s13075-015-0531-7)
Supplement: Additional file 4: Table S4. — Table of assay IDs for specific primer/probe sets (Life Techologies) used for validation of gene expression at AROS Applied Biotechnology A/S. [file 13075_2015_531_MOESM4_ESM.pdf]

Additional table 4

	**Assay genes**	**Assay ID**
1	*Pappa*	Mm01259244_m1
2	*Postn*	Mm00450111_m1
3	*Hapnl1*	Mm00488952_m1
4	*Ano6*	Mm00614693_m1
5	*Il6st*	Mm00439665_m1
6	*Col12a1*	Mm01148576_m1
7	*Crtap*	Mm00517335_m1
8	*Igf1*	Mm00439560_m1
9	*Bmpr1a*	Mm00477650_m1
10	*Bmpr2*	Mm00432134_m1
11	*Inhba*	Mm00434339_m1
12	*Bglap1*	Mm03413826_mH
13	*Ibsp*	Mm00492555_m1
14	*Sparc*	Mm00486332_m1
15	*Gapdh*	Mm99999915_g1
