# Supplementary material for: Kinetics of gene expression and bone remodelling in the clinical phase of collagen-induced arthritis
Source: Arthritis Res Ther. 2015 Mar 5;17(1):43. doi: 10.1186/s13075-015-0531-7 (PMC4391727; doi:10.1186/s13075-015-0531-7)
Supplement: Additional file 8: Table S8. — Correlation data and P-values for correlation between microarray and qPCR data. [file 13075_2015_531_MOESM8_ESM.pdf]

Additional table 7

**Gene**	**R^2**	**R**	**p-value**	
Ano6	0,26	0,51	0,06	*
Bglap1	0,60	0,77	0,0013	**
Bmpr1a	0,05	0,22	0,45	NS”
Bmpr2	0,02	0,15	0,61	NS”
Col12a1	0,57	0,75	0,002	**
Crtap	0,16	0,40	0,15	NS”
Hapln1	0,53	0,73	0,003	**
Ibsp	0,66	0,82	0,0003	***
Igf1	0,46	0,68	0,007	**
Il6st	0,31	0,56	0,04	*
Inhba	0,34	0,58	0,03	*
Pappa	0,25	0,50	0,07	*
Postn	0,72	0,85	0,0001	***
Sparc	0,40	0,63	0,016	*

Data and p-values for the correlation between microarray and qPCR analyses.

* p<0.1, ** p<0.01, *** p<0.001. “NS: Not significant.
